# Risk factors for distant metastasis of dermatofibrosarcoma protuberans

**DOI:** 10.1007/s10195-016-0415-x

**Published:** 2016-06-11

**Authors:** Keiko Hayakawa, Seiichi Matsumoto, Keisuke Ae, Taisuke Tanizawa, Tabu Gokita, Yuki Funauchi, Noriko Motoi

**Affiliations:** 1 Department of Orthopedic Oncology, Cancer Institute Hospital of the Japanese Foundation for Cancer Research, 3-8-31, Ariake, Koto-ku, Tokyo, 135-8550 Japan; 2 Department of Pathology, Cancer Institute Hospital of the Japanese Foundation for Cancer Research, Tokyo, Japan; 3 Division of Pathology, The Cancer Institute, The Japanese Foundation for Cancer Research, Tokyo, Japan

**Keywords:** Dermatofibrosarcoma protuberans (DFSP), Fibrosarcomatous dermatofibrosarcoma protuberans (FS-DFSP), Risk factors, Distant metastasis

## Abstract

**Background:**

Dermatofibrosarcoma protuberans (DFSP) may recur locally but rarely metastasizes. Fibrosarcomatous transformation in dermatofibrosarcoma protuberans (FS-DFSP) is said to have worse prognosis compared with ordinary DFSP (O-DFSP). Since DFSP rarely metastasizes, there have been few reports summarizing data on distant metastasis cases at single institution. The aim of this retrospective study is to review DFSP cases in order to analyze risk factors for metastasis.

**Patients and methods:**

This retrospective study involved 67 patients. We analyzed O-DFSP and FS-DFSP metastasis rates, metastasis sites, time to metastasis, the relationship between frequency of local recurrence and metastasis, and the relationship between primary tumor size and metastasis.

**Results:**

Distant metastasis was found in 5 (7.4 %) of 67 cases with DFSP. Of the five cases, the histopathological diagnosis was FS-DFSP in four cases and O-DFSP in one case. Out of five cases with metastasis, three had not recurred and two had recurred twice. No clear correlation was identified (Fisher’s exact test: *p* = 0.216). The primary tumor diameters in the metastatic cases were 15.0, 12.6, 20.5, 13.0, and 5.0 cm, respectively. The tumor diameters in metastatic cases were significantly larger (Fisher’s exact test: *p* < 0.0001).

**Conclusions:**

In this study, we identified a stronger correlation between DFSP metastasis and tumor size. There was a high possibility that the cases with large tumors might be FS-DFSP, having high rate of metastasis and poor prognosis. In treatment of DFSP, early diagnosis before primary tumor growth and wide resection is considered important.

*Level of evidence* V.

## Introduction

Dermatofibrosarcoma protuberans (DFSP) is a tumor that develops in skin or subcutaneous tissue and is characterized by a protuberant growth pattern [1]. DFSP often recurs locally after surgery, but rarely metastasizes to distant sites and is classified as a sarcoma of intermediate-grade malignancy [2–7]. DFSP sometimes appears with fibrosarcomatous transformation in a subset of tumors, being called fibrosarcomatous dermatofibrosarcoma protuberans (FS-DFSP) [8]. FS-DFSP is said to have a 10–15 % rate of distant metastasis and poorer prognosis compared with ordinary DFSP (O-DFSP) that does not have fibrosarcomatous transformation [9–11]. Therefore, in treatment of DFSP, it is important to accurately evaluate whether O-DFSP contains elements of FS-DFSP.

Since DFSP rarely metastasizes to distant sites, there have been few reports summarizing data about distant metastasis cases at single institution. Therefore, with respect to DFSP, there has been a lack of confirmation about the poor prognostic factors and follow-up methods. In addition, if a patient is diagnosed with FS-DFSP, it has not been determined whether they can be monitored in the same way as a patient diagnosed with O-DFSP.

Therefore, the aim of this retrospective study is to review DFSP (including O-DFSP and FS-DFSP) cases where wide resection of primary tumor was performed in our hospital in order to analyze the relationship between distant metastasis and the following factors: local recurrence, primary tumor size, and fibrosarcomatous transformation, and to compare cases with and without metastasis. Furthermore, we would like to identify appropriate DFSP follow-up methods.

## Patients and methods

This retrospective study involved 67 patients with histopathological diagnosis of DFSP, either O-DFSP or FS-DFSP, who underwent wide resection between January 1977 and July 2013 at the Department of Orthopedic Oncology of our hospital. Of the 67 cases, 50 were male and 17 were female. The mean age was 37.9 years (range 7–70 years). The mean follow-up period was 56.6 months (range 4–263 months).

The histopathological diagnosis before performing wide resection at our hospital was established as follows: 37 cases were diagnosed shortly after resection performed at another hospital, 10 cases were diagnosed at time of recurrence after surgery performed at another hospital, 2 cases underwent incisional biopsy at another hospital, 2 cases underwent excisional biopsy at our hospital, and 16 cases underwent needle biopsy at our hospital.

FS-DFSP was diagnosed according to the criteria of Enzinger and Weiss, which means that FS-DFSP was identified by presence of fibrosarcomatous changes (more than 5 mitoses/10 HPF, “fascicular” growth pattern, increased cellularity, and atypia) in at least 5 % of tumor tissue [3]. Consequently, 7 of the 67 cases were diagnosed with FS-DFSP, and the remaining 60 cases were diagnosed with O-DFSP with resected specimen.

Imaging analysis of primary tumor was conducted using electroradiography or computed radiography. Additionally, computed tomography (CT) scans were used from 1980 onward, and magnetic resonance imaging (MRI) from 1985 onward. Distant metastasis was analyzed using chest X-rays and, from 1980 onward, CT scans. Surgical margin assessment was conducted based on macroscopic and histopathological analyses. Postoperative follow-up observation included clinical assessment and chest X-rays or CT scans for detection of metastasis every 3 months within the first 2 years after surgery and every 6 months from more than 2 to 5 years after surgery.

At the initial visit to the department of our hospital, distant metastasis was noted in 2 (3 %) of the 67 cases; the 2 cases were diagnosed with FS-DFSP. According to the American Joint Committee on Cancer (AJCC) Staging Protocol for Sarcoma of Soft Tissue classification, 46 cases were stage IA, 16 were stage IB, 3 were stage IIC, and 2 were stage IV.

In all cases, surgical treatment was performed for the primary tumor at the department. In cases of initial surgical procedure, the tumor was excised with at least a 1-cm margin of surrounding healthy tissue. In cases treated with surgical resection at another hospital, additional wide resection was performed. As the method for additional wide resection, the original tumor was excised with at least a 1-cm margin of surrounding healthy tissue, including the surgical scar. For cases where it was predicted that the surgical margin could be insufficient or cases where the excised tissue indicated insufficient margin, radiotherapy was used concomitantly. Concomitant radiotherapy was actually administered in 15 cases. No adjuvant chemotherapy was performed.

We analyzed metastasis rates, metastasis sites, time to metastasis, the relationship between frequency of local recurrence and metastasis, and the relationship between primary tumor size and metastasis in DFSP (including O-DFSP and FS-DFSP) cases.

The statistical analysis was conducted using JMP software version 10. Fisher’s exact test was applied to evaluate the relationship between tumor size and metastasis as well as between recurrence and metastasis. The Kaplan–Meier method was used to estimate overall and disease-free survival curves, with differences between groups assessed by log-rank test. *p*-Value <0.05 was considered significant for all statistical analyses.

## Results

Of the 67 DFSP cases, 7 cases were diagnosed with FS-DFSP, and the remaining 60 cases were diagnosed with O-DFSP. Distant metastasis was found in 5 (7.4 %) of 67 cases with DFSP. Of the five cases, the histopathological diagnosis was FS-DFSP in four cases and O-DFSP in one case. With respect to the case of O-DFSP with metastasis, the histological diagnosis of the metastatic lesion was also O-DFSP. Thus, the metastasis rate by histological type was 4 (57 %) of 7 cases for FS-DFSP and 1 (1.7 %) of 60 cases for O-DFSP. Comparing O-DFSP and FS-DFSP, the metastasis rate of FS-DFSP was significantly higher than that of O-DFSP. (Fisher’s exact test: *p* = 0.0002) (Table 2).

Regarding time to metastasis, metastasis was noted as follows: at the initial visit to the department of our hospital in two cases, less than a year after the start of treatment in one case, a year to less than 3 years after the start in one case, and 3 years or less than 5 years in one case, with a mean time of 14.8 months.

At the initial visit, of these five cases, three cases recurred after treatment at another hospital, and two cases were untreated (Table 1). The site of occurrence was neck in one case, anterior chest in two cases, back in one case, and abdomen in one case. Regarding site of initial metastasis, one case had lung metastasis, three had extrapulmonary (intraabdominal, thoracic spine, or axillary lymph node) metastasis, and one had simultaneous metastases to lung and thoracic spine. The outcome in all five cases was death from tumor (Table 1).

**Table 1:** Patients with metastatic tumor

Patient no.	1	2	3	4	5
Age (years) at initial visit	48	49	57	62	37
Sex	M	M	M	M	M
Follow-up period (months)	45	36	22	13	71
Fibrosarcomatous change	+	+	+	+	−
Frequency of recurrence at initial visit	−	−	3	4	1
Tumor size (cm)	15.0	12.6	20.5	13.0	5.0
Metastatic tumor at initial visit	−	−	+	+	−
Metastatic site	Axillary lymph node	Intraperitoneal	Thoracic vertebrae	Thoracic vertebrae, lung	Lung
Time to metastasis (months) after initial treatment	11	15	−	−	41
Clinical outcome	DOD	DOD	DOD	DOD	DOD

*DOD* death of disease

### Frequency of local recurrence and metastasis

**Fig. 1:**
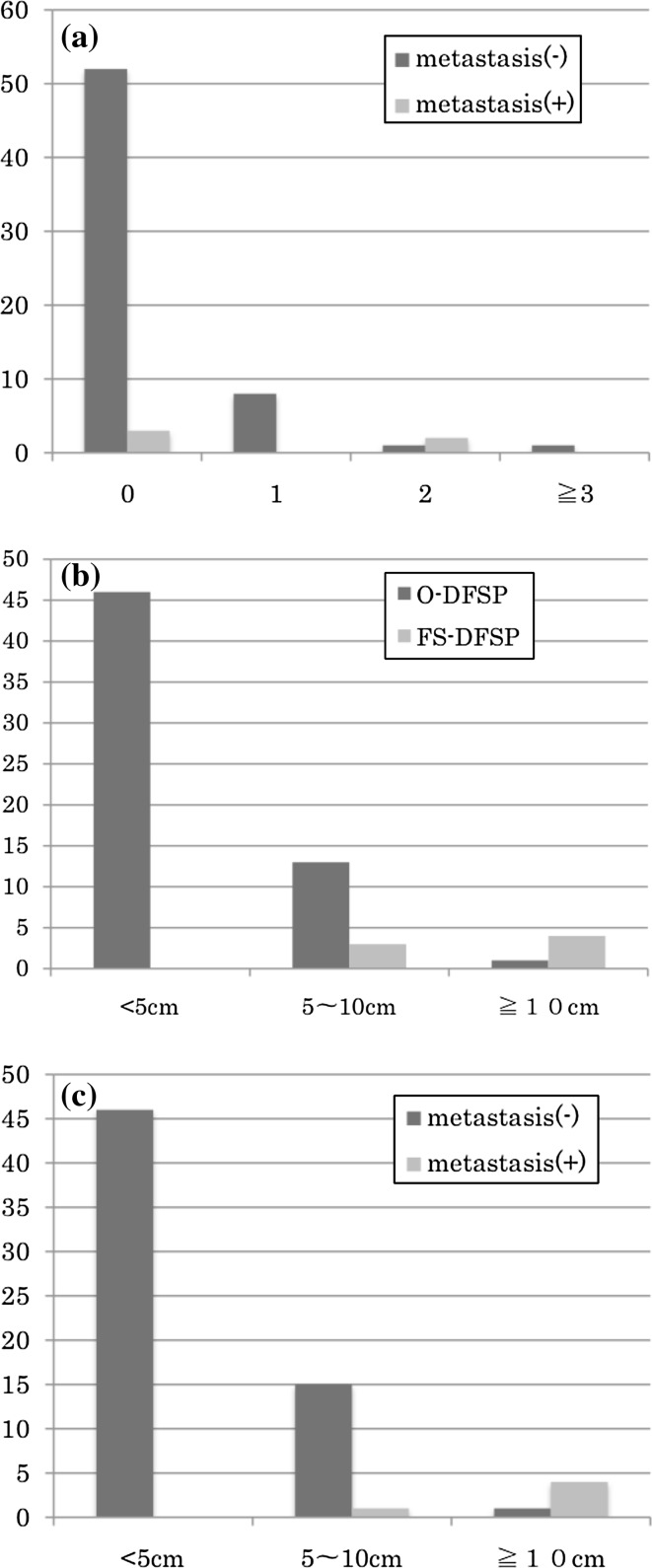
**a** Frequency of local recurrence before visiting our institute. Looking at the frequency of local recurrence and metastasis before visiting our hospital, out of five cases with metastasis, three (60 %) had not recurred and two had recurred twice. In addition, out of 62 cases without metastasis, 52 had not recurred, 8 had recurred once, 1 had recurred twice, and 1 had recurred three times or more. **b** Size of primary tumor. Looking at the 60 cases of O-DFSP, tumor size was less than 5 cm in 46 (77 %) cases, 5 cm to less than 10 cm in 13 (22 %) cases, and 10 cm or larger in 1 (1 %) case. Looking at the seven cases of FS-DFSP, tumor size was 5 cm to less than 10 cm in three (43 %) cases, and 10 cm or larger in four (57 %) cases. **c** Size of primary tumor. Looking at the 62 nonmetastatic cases, tumor size was less than 5 cm in 46 (74 %) cases, 5 cm to less than 10 cm in 15 (24 %) cases, and 10 cm or larger in 1 (2 %) case. Looking at the five metastatic cases, tumor size was 5 cm to less than 10 cm in 1 (20 %) case and 10 cm or larger in 4 (80 %) cases

Regarding frequency of local recurrence and metastasis before visiting our hospital, out of five cases with metastasis, three (60 %) had not recurred and two (40 %) had recurred twice. In addition, out of 62 cases without metastasis, 52 (84 %) had not recurred, 8 (13 %) had recurred once, 1 (1.5 %) had recurred twice, and 1 (1.5 %) had recurred three times or more (Fig. 1a). Concerning local recurrence and metastasis before the initial visit to the department, no clear correlation was identified (Fisher’s exact test: *p* = 0.216) (Table 2).

**Table 2:** Comparing cases with and without metastasis (Fisher’s exact test)

	Metastasis (−)	Metastasis (+)	*p*-Value
O-DFSP	59	1	
FS-DFSP	3	4	0.0002
Recurrence (−)	52	3	
Recurrence (+)	10	2	0.216
<10 cm	61	1	
≥10 cm	1	4	0.0001

Then, considering the relation between local recurrence and metastasis after wide resection performed at the department, 2 (3 %) of 67 cases presented with recurrence. Both of the cases had no metastasis, and there was no clear correlation between recurrence and metastasis.

### Primary tumor size and metastasis

We examined the relationship between tumor size at start of treatment at our hospital and metastasis. In cases where additional wide resection was performed after resection at another hospital, the tumor size before resection at the other hospital was used. Tumor size was less than 5 cm in 46 (69 %) cases, 5 cm to less than 10 cm in 16 (24 %) cases, and 10 cm or larger in 5 (7 %) cases. Looking at the seven cases of FS-DFSP, tumor size was 5 cm to less than 10 cm in three cases, and 10 cm or larger in four cases (Fig. 1b).

The primary tumor diameters in the metastatic cases were 15.0, 12.6, 20.5, 13.0, and 5.0 cm, respectively. Four of the five cases had 10 cm or larger tumors, and all cases with 10 cm or larger tumors were FS-DFSP (Fig. 1c).

We examined the cases with 10 cm or larger tumors. Four (80 %) of the five cases with metastasis had 10 cm or larger tumors, while only 1 (3 %) of the 62 cases without metastasis had a 10 cm or larger tumor. Comparing metastatic with nonmetastatic cases, the tumor diameters in metastatic cases were significantly larger (Fisher’s exact test: *p* < 0.0001) (Table 2).

## Discussion

Dermatofibrosarcoma protuberans (DFSP), first reported in 1890 by Tylar et al. [12], is a cutaneous and subcutaneous tumor that appears commonly on the trunk of patients in their 20s to 50s [13, 14]. FS-DFSP was reported in 1951 by Penner et al. as a case of DFSP metastasis with fibrosarcomatous areas and is a more aggressive tumor than O-DFSP [7, 15–17].

**Fig. 2:**
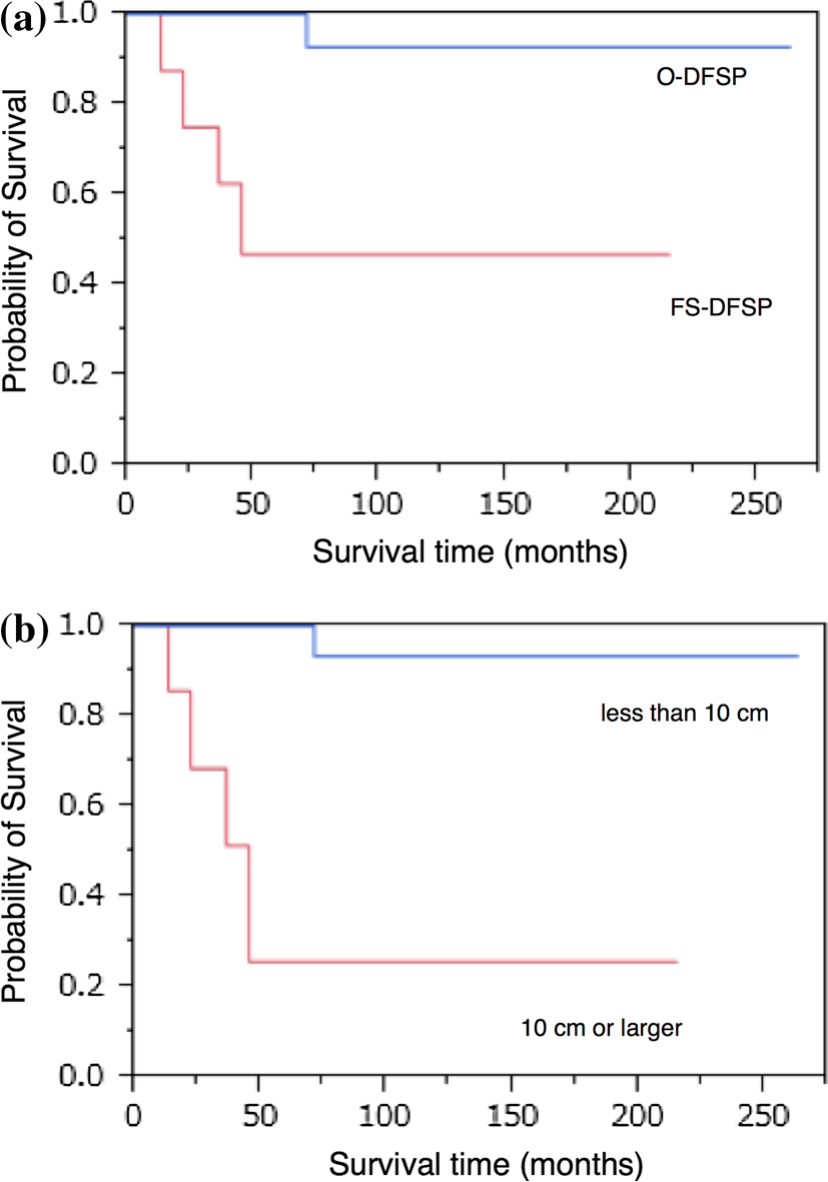
**a** Survival rates of DFSP-O and DFSP-FS. The 5-year cumulative survival rates were 100 % for O-DFSP and 25.7 % for FS-DFSP in our cases, and as also reported by others, the prognosis of FS-DFSP was significantly poor (*p* < 0.0001, log-rank test). **b** Comparison of survival rates between tumors less than 10 cm versus 10 cm or larger. Comparing cases with and without metastasis, cases with metastasis had significantly larger tumor diameter and worse prognosis (*p* < 0.0001, log-rank test)

To diagnose FS-DFSP, a constellation of features is generally used; sarcomatous foci should constitute at least 5–10 % of the tumor. In addition, fibrosarcomatous areas are characterized by higher MIB-1 labeling index [8]. Goldblum et al. reported that FS-DFSP does not have increased risk of poor prognosis compared with O-DFSP. Others reported that the prognosis of FS-DFSP was significantly poor [11, 18–21]. In our study, 5-year cumulative survival rates were 100 % for O-DFSP and 25.7 % for FS-DFSP, and, with similar results to others, the prognosis of FS-DFSP was extremely unfavorable (*p* < 0.0001, log-rank test) (Fig. 2a).

Looking at DFSP metastasis, it has been reported that O-DFSP also metastasizes in rare cases. In 1996, Gloster et al. reported that lung metastasis was observed in 1 (1.1 %) of 89 O-DFSP cases [7]. In 2012, Cai et al. summarized the findings on 260 DFSP cases and reported the O-DFSP metastasis rate as 0.4 % [18]. On the other hand, there have been many reports that FS-DFSP had a metastasis rate of 0–33 %, which is higher than the O-DFSP metastasis rate [10, 11, 18–21] (Table 3).

**Table 3:** Previously reported FS-DFSP study

Study	Number of FS-DFSP	Number of FS-DFSP with metastasis	Rate of metastasis (%)	Average tumor size
John R.	18	0	0	4.2 cm (2–6.8 cm)
Jared R.	41	4	10	4.8 cm (0.4–14.5 cm)
Szollosi Z.	8	1	12.5	4.9 cm (3.5–8 cm)
Mentzel T.	41	5	14.7	6.7 cm (1.5–27 cm)
Cai H.	34	8	23.5	4.5 cm (N/A)
Our study	7	4	57	12.4 cm (5.2–20.5 cm)

*N/A* not available

In 1967, McPeak et al. reported that, in four of the five cases of DFSP with metastasis, the tumor had attained a dimension in excess of 15 cm in diameter [22]. However, there have so far been no reports examining the relationship between tumor size and metastasis. The common DFSP size is thought to be 2–5 cm in diameter [13]. Bowne et al. reported that, out of 159 cases, the sizes were smaller than 5 cm in 134 (84 %) cases, 5 cm to smaller than 10 cm in 21 (13 %) cases, and 10 cm or larger in 4 (3 %) cases [17]. In our 67 cases, the sizes were smaller than 5 cm in 46 (69 %) cases, 5 cm to smaller than 10 cm in 16 (24 %) cases, and 10 cm or larger in 5 (7 %) cases. Four of the five cases with metastasis had 10 cm or larger tumor. Comparing cases with and without metastasis, cases with metastasis had significantly larger tumor size and poorer prognosis (Fig. 2b). The metastasis rates in our cases were 1.7 % for O-DFSP and 57 % for FS-DFSP. Compared with the cases previously reported, the O-DFSP metastasis rates were nearly the same, but the FS-DFSP metastasis rate in our cases was significantly higher. This is because two of seven FS-DFSP cases had distant metastasis at time of first visit and the tumor size of our FS-DFSP cases may have a tendency to be large. Looking at the FS-DFSP cases previously reported, there have been no cases having distant metastasis at first visit. Also, as for the tumor size, while our seven cases of FS-DFSP had a tumor 12.4 cm (range 5.2–20.5 cm) in size on average, FS-DFSP cases previously reported had a tumor 3.5–4.9 cm in size on average, so it could be considered that the cases previously reported tended to have a smaller tumor than our cases [10, 11, 18–21] (Table 3).

Then, regarding recurrence and metastasis, we examined and compared cases with and without metastasis. Compared with O-DFSP, FS-DFSP has been reported to have higher risk of metastasis and recurrence, but no clear correlation between recurrence and metastasis was identified in this study (Fisher’s exact test: *p* = 0.216) (Table 2).

Looking at the metastasis site, Jared et al. reported that, out of four cases of FS-DFSP with metastasis, two were lung metastasis, one was bone metastasis, and one was both lung and bone metastases [10]. In this study, three (75 %) of the four cases of FS-DFSP with metastasis were extrapulmonary metastasis, which indicated that FS-DFSP had a high tendency to metastasize to extrapulmonary sites.

While Abbot et al. reported that the time to metastasis from FS-DFSP was 36–72 months, there have been some cases in which metastasis appeared 10 years or more after initial diagnosis [10]. In our cases, the time to metastasis from FS-DFSP was less than a year in one case and one to less than 3 years in one case. There have been no reports on the time to metastasis from O-DFSP, but in our study, there was a case where the time to the metastasis was 3 years 5 months.

In summary, based on the above, postoperative follow-up methods for DFSP were as follows: In FS-DFSP cases, the rate of distant metastasis was high, so postoperative detection of distant metastasis was essential. Furthermore, since there was a tendency to metastasize to extrapulmonary sites, follow-up examinations such as abdominal CT or ultrasonography were required, taking into account the potential for extrapulmonary metastasis. In O-DFSP cases, follow-up examinations for all cases were not necessary, considering the low rate of distant metastasis of 0.4–1.6 %. However, regular follow-up was recommended for large tumors with potential for FS-DFSP.

In this study, we identified a stronger correlation between DFSP metastasis and tumor size than between metastasis and frequency of recurrence. There was a high possibility that the cases with large tumors might be FS-DFSP, having high rate of metastasis and poor prognosis. In treatment of DFSP, early diagnosis before acceleration of tumor growth and wide resection were important.
